# Reply: Guesstimates are not good enough for determining what is happening in routine care

**DOI:** 10.1038/sj.bjc.6605999

**Published:** 2010-11-09

**Authors:** F H Shabaruddin, R A Elliott, J W Valle, W G Newman, K Payne

**Affiliations:** 1 Department of Health Sciences – Economics, University of Manchester, Manchester M13 9PL, UK; 2 Department of Pharmacy, University of Malaya, Kuala Lumpur 50603, Malaysia; 3 School of Pharmacy, University of Nottingham, Nottingham NG7 2RD, UK; 4 Department of Medical Oncology, Christie NHS Foundation Trust, Manchester M20 4BX, UK; 5 Department of Genetic Medicine, St Mary's Hospital, University of Manchester, Manchester M13 0JH, UK

**Sir,**

We thank Heong *et al* (2010) for their interest in our study on chemotherapy treatment pathways of advanced colorectal cancer (CRC) patients in the United Kingdom (Shabaruddin *et al*, 2010a).

The purpose of our national survey of UK consultant oncologists was to identify predominant treatment pathways within NHS CRC specialties and to determine the extent of variation within current practice. Economic models need to establish current practice as a basis from which to evaluate the incremental cost and benefits of introducing a new intervention, such as the UGT1A1 pharmacogenetic test.

In the United Kingdom, available national data sets do not provide the level of detail needed to describe current clinical practice and the respective outcomes. Some cancer-related registries do exist, such as the UK Association of Cancer Registries and the National Bowel Cancer Audit Programme for CRC surgeries, but do not contain data relevant to current clinical practice in oncology specialties. The National Cancer Intelligence Network (http://www.ncin.org.uk) is currently conducting a chemotherapy data set review that aims to capture data on all chemotherapy carried out in the country. The level of detail for the data collected is yet to be determined. It is necessary to consider that comprehensive UK NHS patient registries are not an easy option. The size of the NHS, funding constraints, issues with data management and quality assurance challenges preclude the establishment and maintenance of such registries for all conditions across the United Kingdom. In an ideal world, information technology in the NHS should allow extraction of data on current practice. This ideal situation does not exist in the current NHS (Anderson, 2010).

In the absence of real-world data on routine care, other means of generating parameters to inform economic analyses are necessary, which may be sufficient depending on the purpose of the evaluation (Cooper *et al*, 2007). Expert estimates are useful starting points to describe current practice and patients’ clinical pathways. It is necessary to elicit expert estimates from across the United Kingdom rather than from one locality in order to understand the variation in routine NHS practice.

Economic models allow the systematic compilation of data from various sources, such as epidemiological studies and clinical trials, within a single framework. Expert estimates are primarily used to inform model parameters in the absence of other data sources. The degrees of uncertainty in the parameters are then explored using the ranges and measures of variation from the expert estimates.

An economic model will allow for exploration of the key areas of uncertainties, generate the expected value of further research to minimise these uncertainties and help inform what research should be conducted to address them. Value of information analysis can indicate whether funding is better directed to more clinical trials or to setting up robust observational studies.

We believe that the usefulness of expert estimates will only be as good as the design, construct and framing of the questions asked. Our final questionnaire was informed by a pilot study of eight consultant oncologists. To address the concern of Heong *et al* regarding the agreement between expert estimates and standard practice, we present (Table 1) preliminary results from an observational study of 48 advanced CRC patients on IrMdG chemotherapy in a UK NHS tertiary care oncology centre (Shabaruddin *et al*, 2010b), in which there were similar parameters with the experts’ estimates (Shabaruddin *et al*, 2010a).

Currently, on the basis of our data, there is no evidence to suggest that pooled UK experts’ estimates varied widely from routine NHS practice. In the absence of other data sources, we believe that expert opinion is useful to inform economic evaluations and to help focus research resources by identifying key areas of uncertainties that offer the best value for future research investments.

Finally, we do agree with Heong *et al* on the need to collect data on current clinical practice and acknowledge the value of access to such data. The key question from the UK perspective is: who will pay and be responsible for collecting, storing and maintaining data on routine NHS clinical practice?

## Figures and Tables

**Table 1:** Expert estimates from 44 NHS consultant oncologists compared with data from an observational study at a UK tertiary care oncology centre

**Parameter**	**Expert estimates**	**Observational study**
Duration of IrMdG chemotherapy	Mean duration 5.0 months	Mean duration 5.0 months (first line 5.2 months, second line 4.7 months)
Incidence of febrile neutropenia (%)	8.4	8.3

Abbreviation: IrMdG=irinotecan (180 mgm^−2^) with infusional 5-FU according to MdG regimen.
